# Apoplastic Nucleoside Accumulation in Arabidopsis Leads to Reduced Photosynthetic Performance and Increased Susceptibility Against *Botrytis cinerea*

**DOI:** 10.3389/fpls.2015.01158

**Published:** 2015-12-23

**Authors:** Manuel Daumann, Marietta Fischer, Sandra Niopek-Witz, Christopher Girke, Torsten Möhlmann

**Affiliations:** Pflanzenphysiologie, Fachbereich Biologie, Technische Universität KaiserslauternKaiserslautern, Germany

**Keywords:** ENT3, NSH3, uridine, adenosine, photosynthesis, *Botrytis cinerea*, *Arabidopsis thaliana*

## Abstract

Interactions between plant and pathogen often occur in the extracellular space and especially nucleotides like ATP and NAD have been identified as key players in this scenario. Arabidopsis mutants accumulating nucleosides in the extracellular space were generated and studied with respect to susceptibility against *Botrytis cinerea* infection and general plant fitness determined as photosynthetic performance. The mutants used are deficient in the main nucleoside uptake system ENT3 and the extracellular nucleoside hydrolase NSH3. When grown on soil but not in hydroponic culture, these plants markedly accumulate adenosine and uridine in leaves. This nucleoside accumulation was accompanied by reduced photosystem II efficiency and altered expression of photosynthesis related genes. Moreover, a higher susceptibility toward *Botrytis cinerea* infection and a reduced induction of pathogen related genes *PR1* and *WRKY33* was observed. All these effects did not occur in hydroponically grown plants substantiating a contribution of extracellular nucleosides to these effects. Whether reduced general plant fitness, altered pathogen response capability or more direct interactions with the pathogen are responsible for these observations is discussed.

## Introduction

Nucleotide metabolism is an essential process in all living organisms as nucleotides function as energy providers, building blocks for nucleic acids, as a signaling component and as precursor for the biosynthesis of the phytohormone cytokinin (Buchanan et al., 2002). Nucleotide metabolism can be roughly divided into three parts: (i) *de novo* synthesis, (ii) salvage of nucleosides and nucleobases, and (iii) catabolism of purines and pyrimidines (Zrenner et al., 2006). Latter requires different subsets of enzymes, for example nucleoside hydrolases (NSH), which initially degrade nucleosides to nucleobases by removing the ribose moiety. Cytosolic NSH1 was attributed a key regulator at the branch point between salvage processes fueling the nucleotide pool and pyrimidine catabolism (Jung et al., 2009). One member of the Arabidopsis NSH protein family, NSH3, belongs to the purine specific IAG-NH family (inosine–adenosine–guanosine nucleoside hydrolases) and was shown to accept adenosine and inosine as substrates (Jung et al., 2011). The results of two independent proteome analyses (Borderies et al., 2003; Kwon et al., 2005) as well as the analysis of apoplastic sap, identified NSH3 as an extracellular protein, assumed to function in extracellular nucleoside turnover (Jung et al., 2011). Incubation of seedlings with jasmonic acid leads to an increased *NSH3* expression, indicating potential participation of NSH3 in plant wound or pathogen response (Taki et al., 2005; Jung et al., 2011).

In addition to catabolic enzymes, specific transport processes are necessary for proper nucleotide metabolism. Whereas several protein families are known to facilitate nucleobase transport (PUPs, NATs, AZGs), the equilibrative nucleoside transporter family (ENT, eight members in *Arabidopsis thaliana*) represents the only group of nucleoside transporters in plants so far (reviewed by Girke et al., 2014). Following a knockout screen of ENT proteins for resistance against toxic 5-fluoro-uridine, the plasma membrane located ENT3 transporter was identified as major nucleoside importer in Arabidopsis (Traub et al., 2007).

So far the origin of extracellular nucleosides is uncertain. They might be liberated by intact or wounded cells, emerge as degradation products of the extracellular messenger eATP (Möhlmann et al., 2014) or can be imported from the rhizosphere (Traub et al., 2007). As both, NSH3 and ENT3 are supposed to participate in shaping the extracellular nucleoside pool, the simultaneous knockout of both genes could be used for the comprehensive investigation of the origin, the role and the physiological functions of extracellular nucleosides.

Extracellular nucleotides (eATP, NAD) function as signals or damage-associated molecular patterns, DAMPS, which i.e., play a role in the plant pathogen response (Zhang and Mou, 2009; Tanaka et al., 2014). To analyze whether nucleosides can exert similar functions, pathogenicity tests are a suitable approach. *Botrytis cinerea* (*B. cinerea*) is a necrotrophic fungus with a broad host range and one of the best studied fungal organisms (Hahn, 2014).

The aim of this work was to provoke an accumulation of apoplastic nucleosides and to study the resulting effects. For this, double knockout plants of the major Arabidopsis nucleoside transporter ENT3 and the extracellular nucleoside hydrolase NSH3 were generated and analyzed. Furthermore, the source of apoplastic nucleosides was investigated by comparing leaf exudates from soil grown with hydroponically grown plants. As extracellular nucleotides and nucleosides are reported to affect pathogen responses of Arabidopsis, a pathogenicity test of the mutants was performed in addition to expression analysis of pathogenicity related genes and measurements of the plants photosynthetic performance.

## Materials and methods

### Plant growth

Wild-type and transgenic *Arabidopsis thaliana* (L.) Heynh. plants (ecotype Columbia) were used throughout. Plants were grown in standardized ED73 (Einheitserde und Humuswerke Patzer, Buchenberg, Germany) soil under short day conditions (120 μmol quanta m^−2^ s^−1^ in a 10 h light/14 h dark regime, temperature 22°C, humidity 60%). Prior to germination, seeds were incubated for 24 h in the dark at 4°C for imbibition (Weigel and Glazebrook, 2002). Growth in Erlenmeyer glass flasks with 100 surface sterilized seed (standard liquid culture) was performed as described by Scheible et al. (2004). Composition of the medium as well as the nitrogen free medium was described earlier Jung et al. (2009). Latter was supplemented with 1 mM inosine. For growth experiments with toxic nucleoside analogs, surface sterilized seeds were sown on half-strength Murashige and Skoog medium (MS, Sigma, Steinheim, Germany) containing 5-fluoro-uridine (Sigma, Steinheim, Germany) and 2-chloro-adenosine (Sigma, Steinheim, Germany) as described earlier (Jung et al., 2011). Growth of plants in hydroponic culture was performed following the description of Conn et al. (2013). Briefly, seeds were placed on microcentrifuge-tube lids containing germination medium (0.75 mM CaCl_2_, 1 mM KCl, 0.25 mM Ca(NO_3_)_2_, 1 mM MgSO_4_, 0.2 mM KH_2_PO_4_, 0.7% agar, pH 5.6) arranged in a 250 ml growth chamber. The insides of the lids were surrounded by liquid germination medium. After stratification as described above, plants were grown under short day conditions (see above) for 7 days. At day 8 the bath solution was adapted to standard nutrient solution over 3 days (0.1 mM CaCl_2_, 2 mM KCl, 2 mM Ca(NO_3_)_2_, 2 mM MgSO_4,_ 0.6 mM KH_2_PO_4_, 1.5 mM NaCl, pH 5.6). At day 21 the plants were transferred to 50 ml centrifuge tubes and placed into 12 L tanks containing standard nutrient solution. All media additionally contained the following micronutrients: 50 μM NaFe(III)EDTA, 50 μM H_3_BO_3_, 5 μM MnCl_2_, 10 μM ZnSO_4_, 0.5 μM CuSo_4_, 0.1 μM Na_2_MoO_3_. Physiological analyses were performed with either 3, 4, or 6 week old plants. In addition to wild-type plants, *ent3-1* (At4g05120; SALK#131585) *nsh3-1* (At5g18860; SAIL#444_C09), and corresponding *ent3:nsh3* double mutants were used (Traub et al., 2007; Jung et al., 2011).

### Mutant screening

Homozygous *ent3:nsh3* mutants were identified in crossed populations of *ent3* and *nsh3* single mutants via PCR using genomic DNA templates. These mutants are characterized by the absence of a corresponding *ENT3* or *NSH3* gene specific PCR product which is obtained in wild-type plants with the primer combinations *ENT3* s/*as* or *NSH3* s/*as* (Supplementary Table 1). In addition, they carry T-DNA insertions in both genes which can be detected by combinations of the SAIL T-DNA specific primer *LB3* and *NSH3* specific primer *NSH3as* and of the SALK T-DNA specific primer *LB335* and *ENT3* specific primer *ENT3s*. To check for the absence of *ENT3* and *NSH3* transcripts in the corresponding double mutants, cDNA was prepared and expression of *ENT3* and *NSH3* was quantified by quantitative RT-PCR as described below with *ENT3* and *NSH3* specific primer pairs (*RT-ENT3, RT-NSH3*). *UBQ10* served as single reference gene in this experiment. All primers used are listed (Supplementary Table 1).

### Biochemical analyses

Isolation of apoplastic sap for enzymatic and metabolite analyses was performed using 6 week old plants as described by Ziegler et al. (2000). Briefly, leaves including the petiole were vacuum infiltrated with 250 mM KCl and subsequently dried using paper. About 20 leaves were arranged in a 5 ml pipette tip with the cut ends facing down and centrifuged in a 15 ml Greiner tube at 350 *g* for 15 min. Immediately after isolation, apoplastic sap was incubated with [^14^C]-radiolabeled inosine for 0/60 min at 30°C. The reaction was terminated by heating the protein to 98°C for 5 min. Thin-layer chromatography on PEI-Cellulose F plates (Merck, Darmstadt, Germany) was used to quantify the amounts of hypoxanthine produced. Separation was performed for 60 min with water, and respective products were detected using a Cyclone Storage phosphorscreen (Perkin-Elmer Life Sciences, Waltham, USA). Spots were quantified using the OptiQuant Software Version 5.0 (Perkin-Elmer Life Sciences, Waltham, USA).

### Transcriptional analyses

For quantitative RT-PCR analyses leaf material of soil or hydroponically grown plants was collected. Depending on the analysis 3 week (photosynthesis) or 6 week (pathogenicity) old plants were used. Directly after harvest, leaf material was ground in liquid nitrogen and 100 mg were used for RNA isolation (RNeasy plant mini kit, Qiagen, Hilden, Germany). To remove any contaminating DNA, samples were treated with DNase (RNase free DNase kit, Qiagen, Hilden, Germany). RNA was quantified and purity was checked photometrically. Total RNA was reverse transcribed with cDNA synthesis kit (qscript cDNA synthesis kit, Quanta Biosciences, Gaithersburg, USA). Quantitative RT-PCR was performed with the IQ SybrGreen supermix (BioRad, Munich, Germany) on a MyIQ PCR Cycler (BioRad, Munich, Germany) using the Primers listed in Supplementary Table 1. Melting curves were checked for all samples. Three biological replicates were measured and for normalization the reference genes *GAPDH, 18SRNA, EF1A*, *UBQ*10, and *ACTIN* (Supplementary Table 1) were included. To qualify for a reference gene in our experiments and on the basis of Remans et al. (2014) the delta C_*t*_ across all experiments must be below 2. This criterion was fulfilled by *UBQ10* and *ACTIN* for pathogenesis related gene analyses and *GAPDH, 18SRNA*, and *ACTIN* for analyses of photosynthesis related genes. Thus, the mean expression value was calculated for each of the genes and the geometric mean was used to normalize gene expression.

### Pathogenicity assay

*B. cinerea* BMM was kindly provided by Prof. Matthias Hahn, University of Kaiserslautern, Germany and grown on HA Medium (10 g l^−1^ malt extract, 4 g l^−1^ glucose, 4 g l^−1^ yeast extract, 15 g l^−1^ agar, pH 5.5) at 20°C for 10 days. Conidiospores were harvested, filtered through glass wool and washed three times with water. Leaves of 6 week old soil or hydroponic culture grown plants were cut off, placed into a humidity saturated chamber and inoculated with suspension containing 1.25 × 10^5^ conidia/ml in Gamborg medium (3 g l^−1^ Gamborg B5 basal (Biochemie BV, Haarlem, Netherlands), 10 mM KH_2_PO_4_, 10 mM Glucose) for pathogenicity and expression analysis. Lesion formation was quantified after 24 h using ImageJ (Schneider et al., 2012). For expression analysis using quantitative RT-PCR pathogenesis related genes were studied. Besides NADPH Oxidase F (*ATRBOHF*, At1g64060) which functions in ROS mediated pathogen response (Chaouch et al., 2012), the Pathogenesis related 1 gene (*PR1*, At2g14610) which is part of the salicylic acid depending pathogen response (Dong, 1998) and *WRKY33* (At2g38470), a transcription factor inducing SA and jasmonate dependent response and a negative regulator of ABA signaling were included (Birkenbihl et al., 2012; Liu et al., 2015). Furthermore, plant defensin 1.2 (*PDF1.2*, At5g44420), a marker for jasmonic acid/ethylene signaling pathway (Ndamukong et al., 2007) encoding an antimicrobial peptide and Jasmonic acid-carboxyl-Methyltransferase (*JMT*, At1g19640) as a key enzyme of jasmonic acid regulated plant response induced by wounding (Seo et al., 2001) and ORA59, which is regulated by *WRKY33* and can induce expression of defense related genes like *PDF1.2* (Pré et al., 2008) were examined.

### Quantification of chlorophyll

For determination of chlorophyll contents, 100 mg frozen tissue was boiled at 95°C for 10 min in 1 ml 80% EtOH. After centrifugation (11,000 g, 5 min) the step was repeated and supernatants were pooled. Chlorophyll contents were determined as absorbance at 652 nm and values corrected according to Arnon (1949).

### Metabolite analyses

For analysis of apoplastic nucleoside composition, apoplastic sap was isolated as described above and immediately used for HPLC quantification in a DIONEX system (P680 HPLC-Pump, ASI-100 Automated Sample Injector, Dionex UVD170U Detektor), UCI-50 Universal Chromatography Interface (Dionex, Sunnyvale, USA) and Nucleodur 100-5 C18 ec-Column (Macherey-Nagel, Düren, Germany). As the eluent, a solution of 10 mM K_2_PO_4_, pH 5.4 and 5.7 mM tetrabutylammonium hydrogen sulfate with gradually increasing (0–80%) acetonitrile, was used. For quantification of sugars, carbonic acids, anions and cations ion chromatography was performed as described by Hassler et al. (2012).

### Pulse-amplitude-modulation (PAM) fluorometry measurements

A MINI-IMAGING-PAM fluorometer (Walz Instruments, Effeltrich, Germany) was used for *in vivo* chlorophyll A fluorescence assays on intact, 3 week old dark-adapted plants. For phenotype quantification, a light induction curve using standard settings was recorded (Schreiber et al., 2007).

### Transport experiments

To test the ability of ENT3 to transport inosine, the open reading frame (ORF) of *ENT3* was cloned into the yeast expression vector pDR196 (Rentsch et al., 1995). Appropriate restriction sites *EcoRI* were introduced with the designed primers *W303*-*ENT3s* and *W303*-*ENT3as* by amplification of *ENT3* from cDNA (Supplementary Table 1). To check for correct cloning the construct was sequenced. Subsequently the construct was transformed into yeast strain W303 (W303; Matα; ura3-1;his3-11; leu2-3_112; trp12; ade2-1; can1-100; YBL042c11,1902::kanMX4) obtained from EUROSCARF (Frankfurt, Germany). Yeast cells transformed with the empty vector pDR196 were used as control in uptake studies. Transport experiments were conducted essentially as described in Wormit et al. (2004) with 10 μM final concentration of [^14^C]-radiolabeled nucleosides.

Arabidopsis gene identifier (AGI) numbers for genes described in this article are: *ENT3*: At4g05120; *NSH3*: At5g18860.

### Statistical analysis

Statistical analyses were performed with One-way ANOVA with Bonferroni post-test GraphPad Prism version 5.00 for Windows, GraphPad Software, San Diego California USA, www.graphpad.com.

## Results

**Figure 1:**
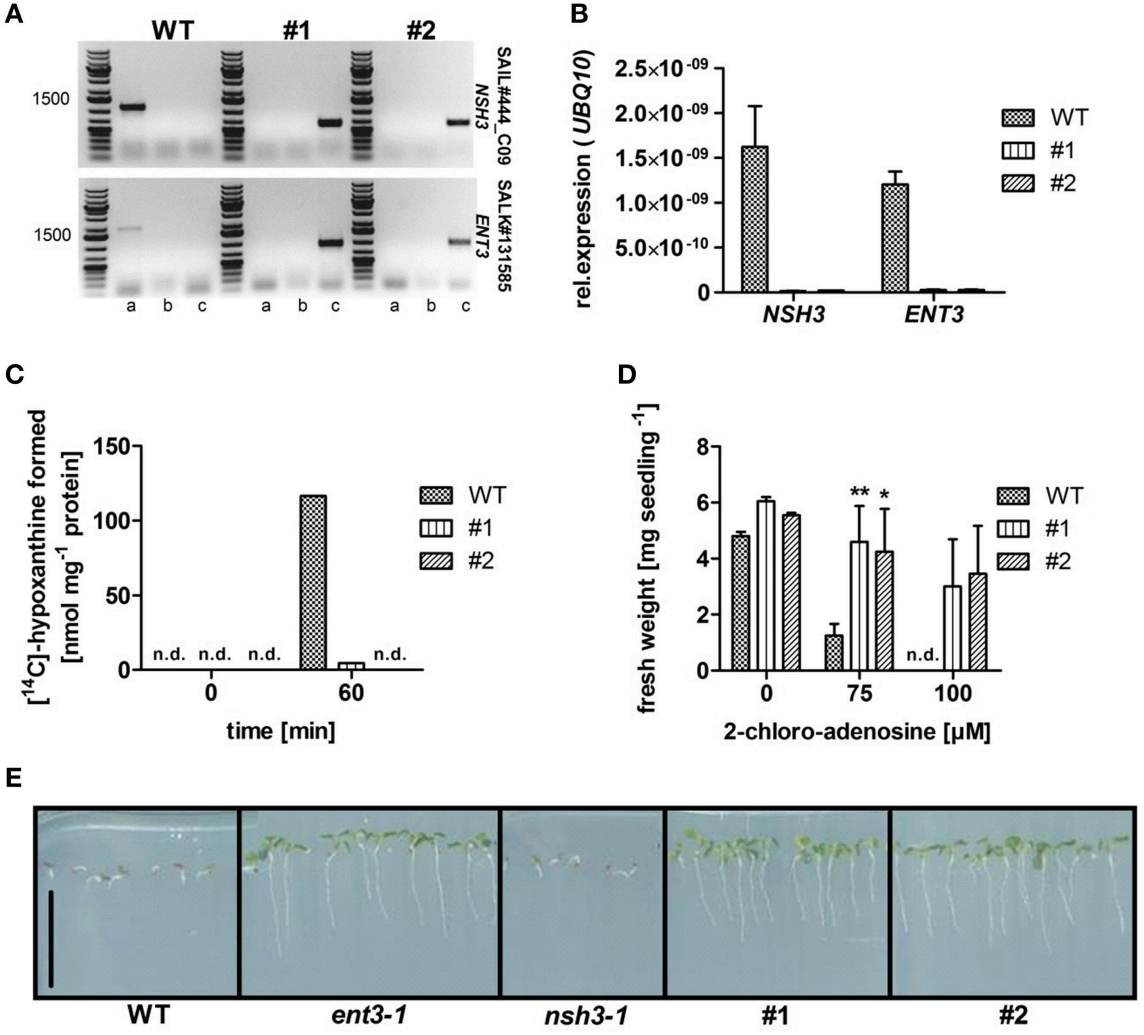
**Characterization of homozygous ***Arabidopsis thaliana*** equilibrative nucleoside transporter 3 (ENT3; SALK_131585) and nucleoside hydrolase 3 (NSH3; SAIL444_C09) double knockout mutants (#1; #2)**. **(A)** PCR analysis on gDNA of wild-type (WT) and homozygous double knockout mutants using gene- (a) and T-DNA-specific primers (b; c) for both possible directions of the T-DNA insertion. **(B)** Analysis of *NSH3* and *ENT3* expression in in WT and *ent3:nsh3* mutants. *UBQ10* expression was used as a reference. Data represent means ± *SE* of three biological replicates. **(C)** Inosine hydrolysis assay using leaf apoplastic sap incubated with [^14^C]-radiolabeled inosine. For zero time point determination samples were heat inactivated immediately after mixing. Experiments were repeated twice with similar results. **(D)** Fresh weight analysis of WT and mutants seedlings grown agar medium containing 2-chloro-adenosine (0, 75, 100 μM) for 7 days. Data represent means ± *SE* of at least 16 biological replicates. Differences between lines were analyzed with One-way ANOVA and a Bonferroni correction at the 95% confidence level. ^*^P < 0.05, ^**^P < 0.005. **(E)** Growth of WT, *ent3-1, nsh3-1*, and *ent3*:*nsh3* (#1, #2) plants grown on agar medium containing 200 μM 5-fluoro-uridine for 14 days. Scale bar 1 cm. n.d., not detectable.

Homozygous T-DNA insertion mutants for equilibrative nucleoside transporter *ENT3* (Traub et al., 2007) were crossed with homozygous T-DNA insertion mutants of the extracellular nucleoside hydrolase *NSH3* (Jung et al., 2011). The resulting heterozygous plants were selfted, a homozygous double mutant isolated and analyzed for the disruption of both genes in a PCR screening based on genomic DNA (Figure 1A). Two *ENT3*-*NSH3* double knockout lines (*ent3:nsh3, #1, #2*) were used for all following experiments. Expression analyses via quantitative RT-PCR confirmed the absence of *ENT3* and *NSH3* transcripts in these mutants (Figure 1B). To support these results, validated biochemical screenings for both mutants were performed (Jung et al., 2011) NSH3 was identified as the sole apoplastic enzyme with activity to convert inosine to the corresponding nucleobase hypoxanthine (Jung et al., 2011). To corroborate the lack of this enzyme activity in *ent3:nsh3* mutants, isolated apoplastic sap was incubated with radiolabeled inosine for 60 min and screened for a conversion to hypoxanthine by thin layer chromatography. Only in extracts from control plants an accumulation of hypoxanthine was determined after 60 min (Figure 1C), clearly indicating the loss of inosine hydrolase activity in *nsh3-1* (Jung et al., 2011) and both *ent3:nsh3* lines. To show blockage of apoplastic catabolism and nucleoside uptake in *ent3:nsh3* plants, resistance toward the cytotoxic adenosine analog 2-chloro-adenosine (CADO) was analyzed. Indeed, after 10 days of growth in the presence of 75 μM CADO, *ent3:nsh3* plants showed almost no reduction in fresh weight (FW), whereas control plants exhibited a marked 74% fresh weight loss (Figure 1D). In a similar screen with 5-fluoro-uridine, the same degree of resistance for both *ent3:nsh3* lines was observed as already before for *ent3-1* (Figure 1E; Traub et al., 2007).

**Figure 2:**
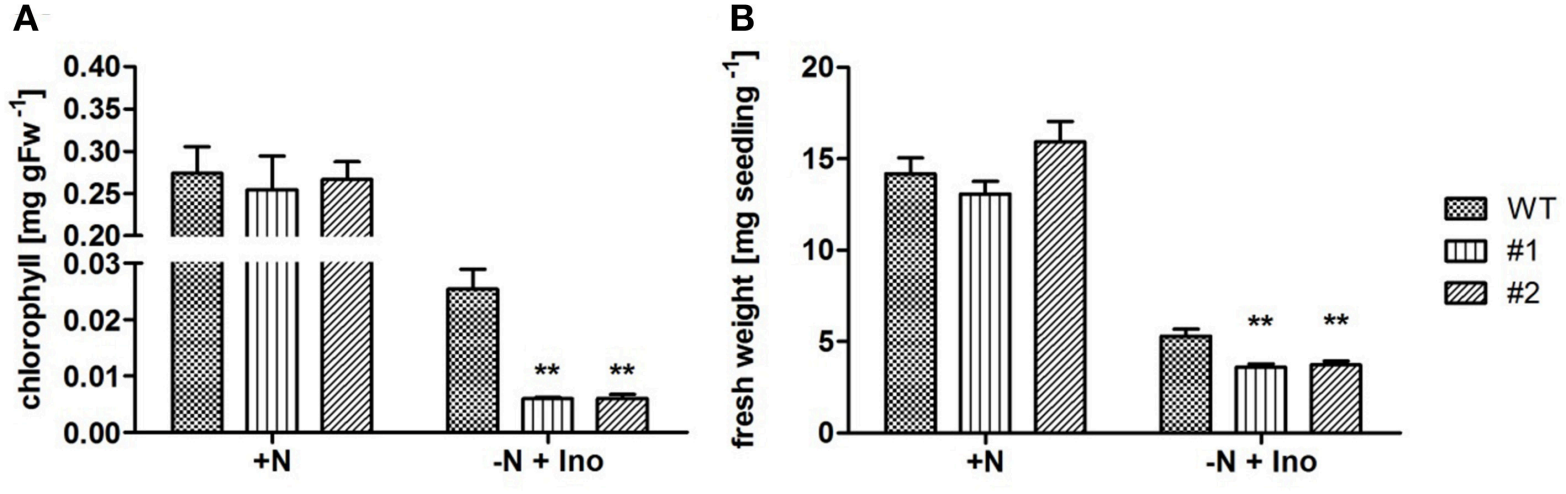
**Effects of inosine feeding on chlorophyll contents and seedling fresh weight production under conditions of limited nitrogen accessibility**. Analysis of chlorophyll contents **(A)** and fresh weight **(B)** of 21 **(A)** and 14 **(B)** day old WT and *ent3:nsh3* plants (#1, #2) grown in standard liquid culture with nitrogen (+N) or nitrogen deprivation but supplemented with 1 mM inosine (−N + Ino). Data represent means ± *SE* of four biological replicates. Differences between lines were analyzed with One-way ANOVA and a Bonferroni correction at the 95% confidence level. ^**^P < 0.005.

Growth of *ent3:nsh3* mutants on soil or standard MS agar plates revealed no differences in development compared to controls. This observation is supported by unchanged chlorophyll levels of seedlings grown in liquid culture under standard conditions (Figure 2A). However, when inosine, which serves as a substrate for both ENT3 (Supplementary Figure 1) and NSH3 (Jung et al., 2011) was present as sole nitrogen source, chlorophyll levels of both mutants were reduced more than 75% (#1: 0.00596 mg gFW^−1^; #2: 0.00602 mg gFW^−1^) compared to controls (WT: 0.0254 mg gFW^−1^; Figure 2A). These results were also reflected in the fresh weights of the analyzed mutants where inosine as sole nitrogen source promoted growth of control plants to 5.3 mg seedling^−1^ whereas *ent3:nsh3* #1 exhibited 3.6 mg seedling^−1^ and *ent3:nsh3* #2 3.7 mg seedling^−1^ (Figure 2B). In contrast, adenosine or uridine provided as sole nitrogen source did not promote seedling growth in any of the lines.

To obtain a more complete picture about physiological processes in the mutants, 4 week old soil and hydroponically grown plants were monitored for soluble sugar, anion, cation and carboxylic acid contents in leaves. Even if metabolite contents differed with regard to growth conditions, no changes were detectable within the single plant lines (Supplementary Figure 2). Only in case of citric acid significant differences were observed. In both soil grown *ent3:nsh3* lines citrate levels were increased 30 and 42% above control levels (Supplementary Figure 2). This effect could not be detected for plants grown in hydroponic culture.

**Figure 3:**
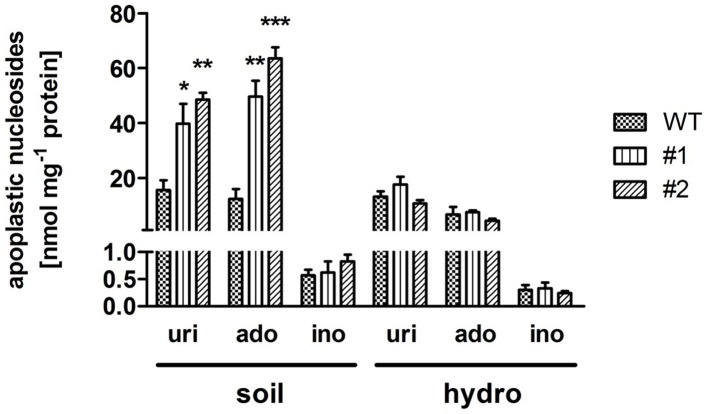
**Uridine, adenosine, and inosine contents in isolated apoplastic extracts**. Plants were grown under ambient conditions on soil (soil) or hydroponic culture (hydro) for 6 weeks before nucleoside composition of the apoplastic extract was determined by high performance liquid chromatography. uri, uridine; ado, adenosine; ino, inosine. Data represent means ± *SE* of three biological replicates. Differences between lines were analyzed with One-way ANOVA and a Bonferroni correction at the 95% confidence level. ^*^P < 0.05; ^**^P < 0.005, ^***^P < 0.001.

It could be shown that *ent3:nsh3* plants are impaired in uptake and catabolism of supplied nucleosides. Therefore, one can assume an accumulation of these substances in the apoplastic space. To test this, apoplastic sap was collected from mature leaves and analyzed by HPLC. Clearly, uridine (uri, #1: 2.56-fold; #2: 3.13-fold) and adenosine levels (ado, #1: 3.49-fold; #2: 4.48-fold) increased in apoplastic sap from *ent3:nsh3* plants compared to controls (Figure 3). In contrast to soil grown plants, hydroponically grown plants cultivated in media lacking any organic compounds did not show accumulation of nucleosides in apoplastic fluid above control levels (Figure 3).

**Figure 4:**
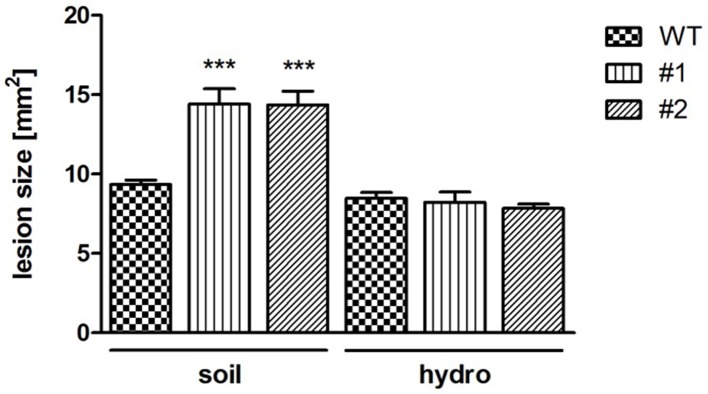
*****Botrytis cinerea*** BMM infection caused lesion formation**. Lesion formation after inoculation with *B. cinerea* BMM conidiospores at *ent3:nsh3* double knockout plants grown on soil (soil) and hydroponic culture (hydro) was monitored after 24 h. Data represent means ± *SE* of at least 64 (soil) and 31 (hydro) biological replicates. Differences between lines were analyzed with One-way ANOVA and a Bonferroni correction at the 95% confidence level. ^***^P < 0.001.

*NSH3* expression was shown to increase after wounding or jasmonic acid (JA) treatment (Jung et al., 2011). Like wounding, also the response to necrotrophic pathogens like *Sclerotinia sclerotiorum* (Wang et al., 2015) and *B. cinerea* (Aubert et al., 2015) can induce JA signaling pathways. As a consequence, pathogenicity tests were performed with the necrotrophic fungus *B. cinerea* BMM, as this was shown to be well-suited for Arabidopsis infection (Zimmerli et al., 2001). For this, droplets of conidiospore suspensions were spotted onto leaves cut from 6 week old healthy plants. Subsequently, the leaves were incubated in a humid chamber and lesion formation was quantified 24 h after application of spores. Double mutant *ent3:nsh3* lines exhibited a 1.5-fold increase in mean lesion size in comparison control leaves (Figure 4). When the same experiment was performed with hydroponically grown plants, no alterations in lesion size formation were observed between the lines after 24 h (WT: 8.47 ± 0.37 mm^2^; #1: 8.23 ± 0.64 mm^2^; #2: 7.84 ± 0.28 mm^2^).

**Figure 5:**
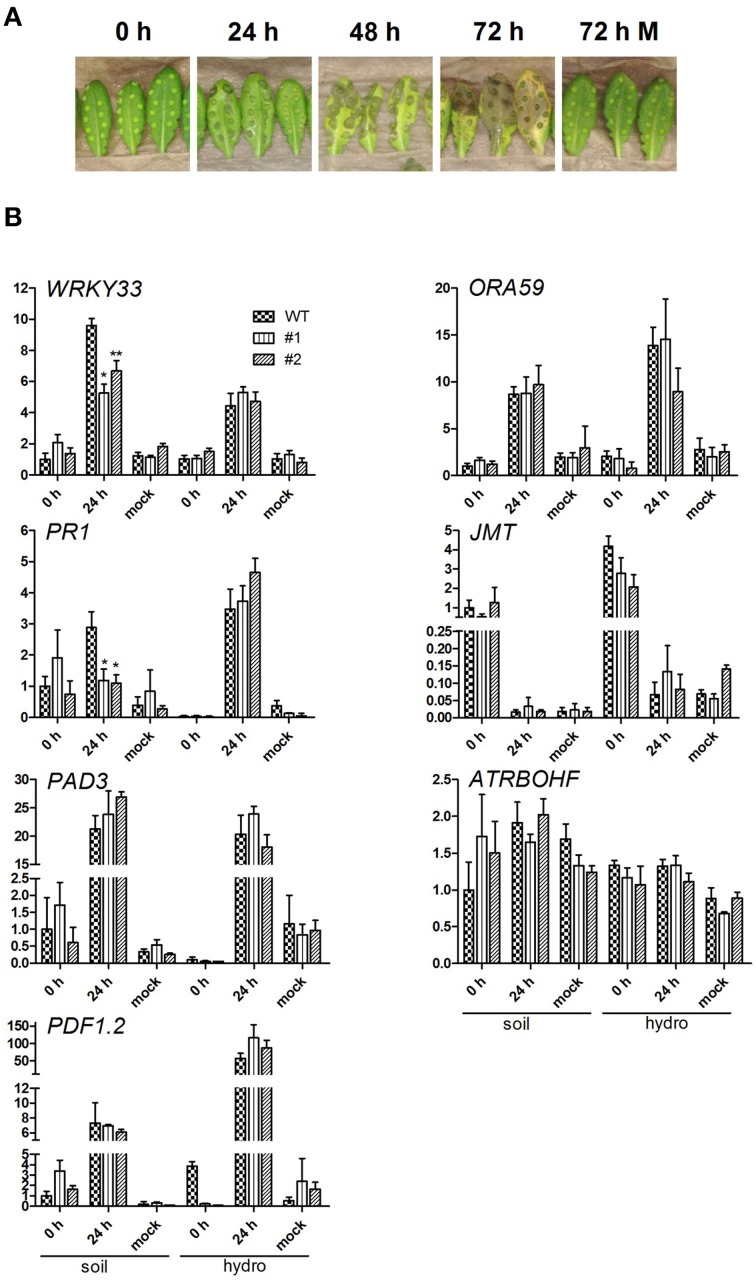
*****Botrytis cinerea*** infection and expressional analysis of pathogenesis related transcripts**. **(A)** Representative infection study using *B. cinerea* BMM condiospores infecting 6 week old, soil grown wild-type plants. The infection studies were performed using WT and both *ent3:nsh3* double knockout lines grown on soil and hydroponic culture. Conidiospore and mock inoculated leaves were documented 0 and 24 hpi before used for RNA extraction. **(B)** For quantitative RT-PCR analysis of *WRKY33, PR1, PAD3, PDF1*.*2, ORA59, JMT*, and *ATRBOHF* in the context of *B. cinerea* BMM infection, five biological replicates per condition were used. The expression was normalized using the geometric meridian of the housekeeping genes *EF1*α and *UBQ10* and represents the relative expressional change compared to respective expression of WT plants grown on soil 0 h after infection. Differences between lines were analyzed with One-way ANOVA and a Bonferroni correction at the 95% confidence level. ^*^P < 0.05; ^**^P < 0.005.

As necrotrophic pathogens rely on killing host cells to receive nutrients for growth, plants developed several defense strategies. Thus, resistance of Arabidopsis against necrotrophs like *B. cinerea* appears to be under complex genetic control (Rowe and Kliebenstein, 2008). This results in tremendous reprogramming of the host transcriptome (AbuQamar et al., 2006), including key regulators like transcription factors. Thus, transcripts of defense related genes were quantified in leaves challenged with *B. cinerea* using quantitative RT-PCR. Control leaves were analyzed 0 h after infection and after 24 h treated with buffer alone (Mock; Figure 5). The detection of Botrytis *ACTIN* transcripts confirmed the successful infection of all analyzed leaves 24 hpi, but was absent in Mock treated samples (data not shown).

All examined genes except for *ATRBOHF* and *JMT* showed increased expression levels in leaves of soil and hydroponically grown plants after pathogen treatment indicating the activated plant response to pathogen challenge (Figure 5B). In line with this, no significant change in transcript levels of all analyzed genes could be detected between 0 h control and 24 h Mock conditions. Transcript levels for regulators of the JA coordination and ethylene (ET) defense *(ORA59*, McGrath et al., 2005), redox homeostasis (*ATRBOHF*, NADPH oxidase, Chaouch et al., 2012), JA response (*JMT*, JA-Carboxyl-Methyltransferase, Seo et al., 2001; *PDF1.2*, plant defensin 1.2, Meng et al., 2013) and phytoalexin synthesis (*PAD3*, Ferrari et al., 2007) did not show significant differences between WT and mutant lines. This finding is independent of the plant growth conditions. The most marked differences in expression between wild-type and mutant lines were observed for *WRKY33* and *PR1* (pathogenicity related protein 1, Dong, 1998; Spoel et al., 2003) 24 hpi in leaves of soil grown plants (Figure 5B). In both cases the mutants showed significantly reduced expression. Although in wild-type and mutant plant leaves an increased *WRKY33* expression was detected compared to control conditions (WT: 9.59-fold), the increase was less pronounced in both mutant lines (#1: 2.53-fold; #2: 4.86-fold). *PR1* expression was not significantly altered 24 hpi in mutants compared to both control conditions, while the transcript increased in wild-type plant leaves 2.9-fold above the 0 h level (Figure 5B). No expressional alterations were detected in hydroponically grown lines (Figure 5B). WRKY33 was identified as a superior regulator during challenge with *B. cinerea*, involved in modulation of JA- and ET-mediated defense mechanisms, salicylic acid (SA) signaling and of redox homeostasis genes (Zheng et al., 2006; Birkenbihl et al., 2012). The data clearly indicates, that at preinfectious stages the transcripts of the examined pathogenesis related genes in soil as well as hydroponically grown plants do not differ *per se*. Thus, the significantly changed expression of *WRKY33* and *PR1* depends on the growth condition.

**Figure 6:**
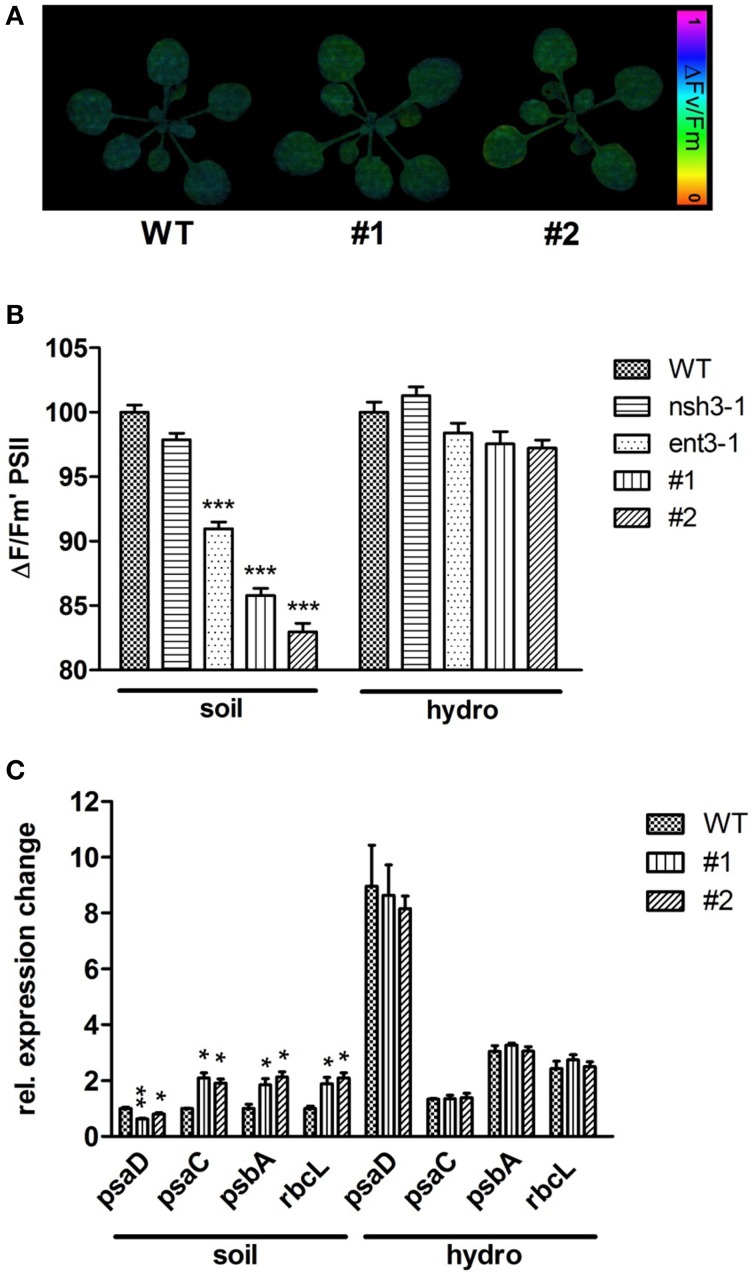
**Photosynthetic efficiency and expressional analysis of photosynthesis related genes**. **(A)** PSII efficiency of 3 week old WT, *ENT3*, and *NSH3* single and double knockout mutants grown on soil and hydroponic culture was examined using a MINI-IMAGING-PAM fluorometer (Walz Instruments, Effeltrich, Germany). Altered PSII efficiencies (ΔFM/Fm′) were indicated by color changes. **(B)** Quantification of PSII efficiency. Data represent means ± *SE* of at least 10 plants. Differences between lines were analyzed with One-way ANOVA and a Bonferroni correction at the 95% confidence level (^***^P < 0.001). **(C)** Expressional analysis of photosynthesis related genes using quantitative RT-PCR. The expression of *PSAC, PSAD, PSBA* and *RBCL* was normalized using the geometric meridian of the housekeeping genes *GAPDH, ACTIN*, and *18SRNA*. Data represent means ±SE of three biological replicates. Differences between lines were analyzed with One-way ANOVA and a Bonferroni correction at the 95% confidence level. ^*^P < 0.05; ^**^P < 0.005.

To elucidate further effects of the extracellular accumulation of nucleosides in *ent3:nsh3* mutants, photosynthetic processes, particularly the photosynthetic performance as photosystem II efficiency (ΔFM/Fm′) was analyzed (Figures 6A,B). A reduced PSII efficiency could be observed for *ent3-1* (−9.1%). In *ent3:nsh3* mutants this value was even more decreased by 15% (#1) and 18% (#2) when grown on soil (Figure 6B). In contrast, mutant plants grown in hydroponic culture exhibited no alterations of PSII efficiency in comparison to control plants (Figures 6A,B). When monitoring the expression pattern of twelve nuclear or plastid encoded photosynthesis related genes, it turned out that transcripts of *PSAC* (#1: 2.1-fold; #2: 1.91-fold), *PSBA* (#1: 1.85-fold; #2: 2.12-fold) and *RBCL* (#1: 1.89-fold; #2: 2.09-fold) were specifically upregulated in soil grown mutants, but not in wild-types or hydroponically grown mutants (Figure 6C). For *PSAD* a significant decreased transcript level could be detected for both soil grown mutants (#1: 0.63-fold; #2: 0.80-fold, Figure 6C). All other genes analyzed did not exhibit significant expression differences (Supplementary Figure 3).

## Discussion

### Double knockouts of *ENT3* and *NSH3* show accumulation of extracellular nucleosides

Research over the last years identified nucleosides as valuable metabolic intermediates involved in different aspects of plant metabolism (Jung et al., 2009, 2011; Cornelius et al., 2012; Girke et al., 2014). Here, we describe the generation of a mutant lacking the main nucleoside import activity as well as extracellular nucleoside hydrolase activity. This *ent3:nsh3* double mutant combines characteristics of corresponding single mutants as increased resistance against the toxic purine and pyrimidine nucleoside analogs 2-chloro-adenosine and 5-fluoro-uridine, a diminished capacity to degrade extracellular purine nucleosides and a reduced ability to grow on inosine as sole nitrogen source. Inosine is a preferred substrate for ENT3 and NSH3. For white spruce embryos it was shown that inosine, in contrast to adenosine, is catabolized almost completely (Ashihara et al., 2001). These results are in line with a lower activity of NSH3 and cytosolic NSH1 for adenosine compared to inosine (Jung et al., 2009, 2011). As inosine cannot be converted to xanthosine directly (Dahncke and Witte, 2013), it first has to become deribosylated to hypoxanthine either in the apoplast by NSH3 or after import via ENT3 by NSH1 to become further catabolized to xanthine by xanthine dehydrogenase (Hesberg et al., 2004). Apoplastic hypoxanthine can subsequently be imported by plasma membrane located NAT3 and NAT12 transport proteins (Niopek-Witz et al., 2014). When direct import and extracellular catabolism are not active in *ent3:nsh3* mutants, inosine cannot function as efficient nitrogen source. Whereas uridine and adenosine accumulated in apoplastic leaf extracts of soil grown mutant plants two- to five-fold above the level of control plants, inosine levels were not altered. Therefore, one can assume that at least quantitatively inosine plays a minor role in nucleotide metabolism in Arabidopsis leaves.

*Ent3:nsh3* mutants were used as a tool to dissect the effect of reduced nucleoside import accompanied by apoplastic nucleoside accumulation. Nucleosides can either be taken up from the soil (Traub et al., 2007; Cornelius et al., 2012) where they can appear in respectably high amounts (Phillips et al., 1997) or originate from liberation within the plant. When comparing apoplastic nucleoside levels from soil grown plants with plants of the same size grown in fully anorganic hydroponic solution, two observations could be made: First, control plants show a similar, basal accumulation of nucleosides under both conditions. As the apoplastic fluid was free of contaminating cytosolic marker enzyme (glyceraldehyde-3-phosphate dehydrogenase) activity (data not shown), these nucleosides are not regarded as contaminations but as originating from endogenous sources. Second, the marked increase of apoplastic nucleosides in soil grown *ent3:nsh3* mutants was not observed in hydroponically grown plants. This supports the view that at least parts of the extracellular nucleosides might originate from the rhizosphere when plants are grown on soil. We aimed to test this hypothesis by feeding nucleosides to hydroponic culture and observed fast bacterial growth in the (otherwise anorganic) medium. Sterile growth on agar plates represents an alternative strategy. However, sterile culture is so much different from the applied soil and hydroponic culture and furthermore infection assays and photosynthesis measurements are hardly possible. Therefore, we decided not to perform sterile culture experiments and postpone the clarification of the origin of apoplastic nucleosides to future studies.

### Physiological consequences of extracellular accumulation of nucleosides—photosynthetic efficiency

As a general measure of plant fitness the photosynthetic efficiency of *ent3:nsh3* mutants and controls was determined as ΔFM/Fm′. Clearly, PSII efficiency was significantly higher under conditions where nucleosides could be imported i.e., in control plants. Such positive effects of supplied nucleosides were also observed for *Ricinus communis* cotyledones where adenosine supplementation supported seedling growth (Flörchinger et al., 2006). Restricted NSH3 activity alone does not lead to reduced PSII efficiency, only when ENT3 is missing at the same time this effect can be observed. Therefore, co-limiting adenosine cleavage in addition to omitted import seems to be causative for the observed reduction in PSII efficiency. As uridine is no substrate for NSH3, adenosine is probably the most effective metabolite in these experiments.

The reduced PSII efficiency is not due to a general downregulation of photosynthesis related transcripts as known for the biotic stress response (Bilgin et al., 2010; Windram et al., 2012). In contrast, *PSAC, RBCL*, and *PSBA* transcripts, all plastid encoded, were significantly more abundant in soil grown mutants whereas nuclear encoded *PSAD* transcripts were lowered. It is therefore unlikely that reduced PSII efficiency is caused by transcriptional downregulation of corresponding genes. It is more likely that other, so far unknown effectors act on photosynthesis and the observed plants response might be a compensation reaction. In the typical response to pathogen challenge downregulation of photosynthetic gene expression is assumed to enable the plant to reallocate nitrogen resources for synthesis of new defense proteins (Windram et al., 2012). As *ent3:nsh3* mutants do not show altered transcript levels of photosynthesis related genes *per se*, a regulation different from the pathogen response pathways is more likely.

Reduced PSII efficiency was not reflected in changes in sugar, anion, or cation contents on a whole leaf basis. However, among the carboxylic acids, citrate levels increased slightly but significantly in double mutant plants. A generally repressive effect of citrate on the abundance of photosynthetic transcripts was shown (Finkemeier et al., 2013). However, whether this links increased citrate levels to reduced PSII efficiency in our mutants remains elusive so far.

### Physiological consequences of extracellular accumulation of nucleosides—response to *B. cinerea* challenge

Extracellular metabolites such as extracellular ATP (eATP) and NAD have been identified as danger signals or elicitors and play a role in the plant response to pathogen attack (Zhang and Mou, 2009; Cao et al., 2014; Tanaka et al., 2014). In addition, extracellular adenosine was shown to attenuate ATP induced generation of reactive oxygen species (Song et al., 2006). Therefore, we analyzed the response of *ent3:nsh3* mutants toward infection with the necrotrophic, highly virulent *B. cinerea* BMM strain. Lesion formation significantly increased in both *ent3:nsh3* mutants when grown on soil. However, when plants were grown hydroponically where no nucleosides accumulate in the apoplastic space and no difference in PSII efficiency was observed, also lesion formation was indistinguishable between mutants and control plants. This is indicative of a function of extracellular nucleosides in plant response toward *B. cinerea* infection. In line with this, expression of *NSH3* was shown to increase after wounding or treatment with JA, a known signaling compound in necrotrophic pathogen attack. The expression of genes involved in different plant pathogen response pathways showed no differences between mutant and control lines at time 0 h or Mock treatment, indicating that extracellular nucleosides themselves are not perceived as danger signals or DAMPs (damage associated molecular patterns). Exogenously administered ATP similarly does not induce *PR1* expression, whereas NAD(P) does (Zhang and Mou, 2009). Obviously, nucleosides and purine nucleotides exert different effects as pyridine nucleotides.

The expression of *ATRBOHF, PAD3, ORA59*, and *PDF1.2* increased upon pathogen treatment in soil and hydroponically grown plants, but remained indistinguishable between mutants and controls. However, the increase in *PR1* and *WRKY33* transcripts in *ent3:nsh3* soil grown plants accounted only to 50% of the transcript increase measured in wild-type plants. Interestingly, reduced *WRKY33* expression in knockout lines causes decreased pathogen resistance and an elevated numbers of dying cells after *B. cinerea* inoculation for 40 h (Birkenbihl et al., 2012). Furthermore, conidiophores appeared already 3 dpi only on *WRKY33* knockout plants but not on WT plants. This indicates the outstanding regulatory function of WRKY33 on the plant response during pathogen challenge. It is easy to assume, that altered expression of this key player in *ent3:nsh3* knockout plants could also be causative for increased pathogen expansion, indicated by enlarged lesion formation. It is further known that WRKY33 binds to *PAD3* promotor regions after being released from a trimeric complex (Andreasson et al., 2005; Qiu et al., 2008) regulating the synthesis of the phytoalexin camalexin, a critical component for plant defense mechanisms. Mutants deficient in PAD3 are highly susceptible to *B. cinerea* (Ferrari et al., 2007). Furthermore, ORA59, a transcription factor of the AP2-ERF family, is involved in the regulation of JA and ET defense genes (McGrath et al., 2005) and is positively regulated by WRKY33 (Birkenbihl et al., 2012). Also the expression of *PDF1.2* depends on the presence of WRKY33. These examples additionally highlight the outstanding role of the WRKY transcription factor and its broad regulatory functions in pathogen response pathways. Although there is no respective response for *PAD3, ORA59*, and *PDF1.2* expression in our experiments as seen for WRKY33 knockout plants (Birkenbihl et al., 2012), it can be suggested, that the alteration of *WRKY33* transcript levels has wide spreading influences on general pathogen response pathways.

Besides influences on *ATRBOHF, PAD3, ORA59*, and *PDF1.2* expression, it was shown that WRKY33 negatively regulates synthesis of SA, a major signaling component critical for the development of systemic acquired resistance and the induction of PR genes (Malamy et al., 1990; Métraux et al., 1990). Thus, in *WRKY33* knockout mutants a higher *PR1* expression could be detected resulting in three times higher SA accumulation 24 h after infection with *B. cinerea*. This means that WRKY33 is involved in preventing the inappropriate induction of the SA pathway during the challenge with necrotrophic pathogens. This alteration of transcript can't be seen in our experiments as reduced *WRKY33* expression is accompanied with decreased *PR1* transcript levels, pointing to a reduced SA dependent defense reaction. As WRKY33 is also involved in JA induced plant defense, JA dependent signaling might be compromised in *ent3:nsh3*. This observation is in line with the described increased expression of *NSH3* by JA (Jung et al., 2011).

The *ent3:nsh3* mutant response at the level of gene expression (photosynthesis related genes and pathogenesis related genes) differs from the normal response toward biotic stress and points to an interference with related signaling cascades. Supporting results for this view come from the analysis of the OXT1 mutant exhibiting altered adenine phosphoribosyltransferase 1. In this mutant moderately increased cellular adenine contents (1.5-fold) lead to markedly increased resistance against oxidative stress (Sukrong et al., 2012). Thus, altered (purine) nucleobase or nucleoside levels may act as physiological signals affecting stress responses in Arabidopsis. Adaptation of ENT3 and NSH3 activity to environmental conditions are obviously required to balance nucleobase and nucleoside levels to allow for optimal stress response. In animals, both ATP and adenosine are known to function as extracellular signaling molecules. A plant receptor for eATP has been identified recently with DORN1 (Choi et al., 2014) whereas receptors for adenosine, other nucleosides or nucleobases are unknown so far. However, DORN1 is a member of plant specific lectin receptor kinases which comprise a large gene family. Whether any lectin receptor kinase recognizes nucleosides or nucleobases has to be shown in future experiments.

## Author contributions

Conceptualization, TM; Methodology, MD; Investigation, MD, MF, SN, CG, Writing-Original Draft, TM; Writing-Review and Editing, MD, CG.

### Conflict of interest statement

The authors declare that the research was conducted in the absence of any commercial or financial relationships that could be construed as a potential conflict of interest.
