# Supplementary material for: Apoplastic Nucleoside Accumulation in Arabidopsis Leads to Reduced Photosynthetic Performance and Increased Susceptibility Against Botrytis cinerea
Source: Front Plant Sci. 2015 Dec 23;6:1158. doi: 10.3389/fpls.2015.01158 (PMC4688390; doi:10.3389/fpls.2015.01158)
Supplement: Supplementary file 1 [file Image1.PDF]

Supplementary Material

Apoplastic nucleoside accumulation in Arabidopsis leads to reduced photosynthetic performance and increased susceptibility against *Botrytis cinerea*.

Manuel Daumann, Marietta Fischer, Sandra Niopek-Witz, Christopher Girke, Torsten Möhlmann*

*** Correspondence:** Corresponding Author: Dr. Torsten Möhlmann

email: moehlmann@biologie.uni-kl.de

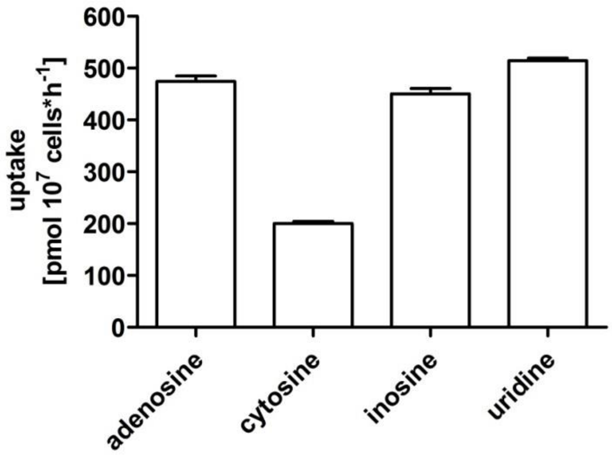

Supplementary Figure 1. Uptake of [^{14}C] purine and pyrimidine nucleosides into intact *Saccharomyces cerevisiae* cells expressing *ENT3*. Cells constitutively expressing *ENT3* were incubated for 1 min at 10 µM final substrate concentration. Uptake of Yeast cells harboring the empty vector (pDR196) were substracted. Data represent means ±SE of three biological replicates.
