# Supplementary material for: Apoplastic Nucleoside Accumulation in Arabidopsis Leads to Reduced Photosynthetic Performance and Increased Susceptibility Against Botrytis cinerea
Source: Front Plant Sci. 2015 Dec 23;6:1158. doi: 10.3389/fpls.2015.01158 (PMC4688390; doi:10.3389/fpls.2015.01158)
Supplement: Supplementary file 2 [file Image2.PDF]

Supplementary Material

Apoplastic nucleoside accumulation in Arabidopsis leads to reduced photosynthetic performance and increased susceptibility against *Botrytis cinerea*.

Manuel Daumann, Marietta Fischer, Sandra Niopek-Witz, Christopher Girke, Torsten Möhlmann*

*** Correspondence:** Corresponding Author: Dr. Torsten Möhlmann

email: moehlmann@biologie.uni-kl.de

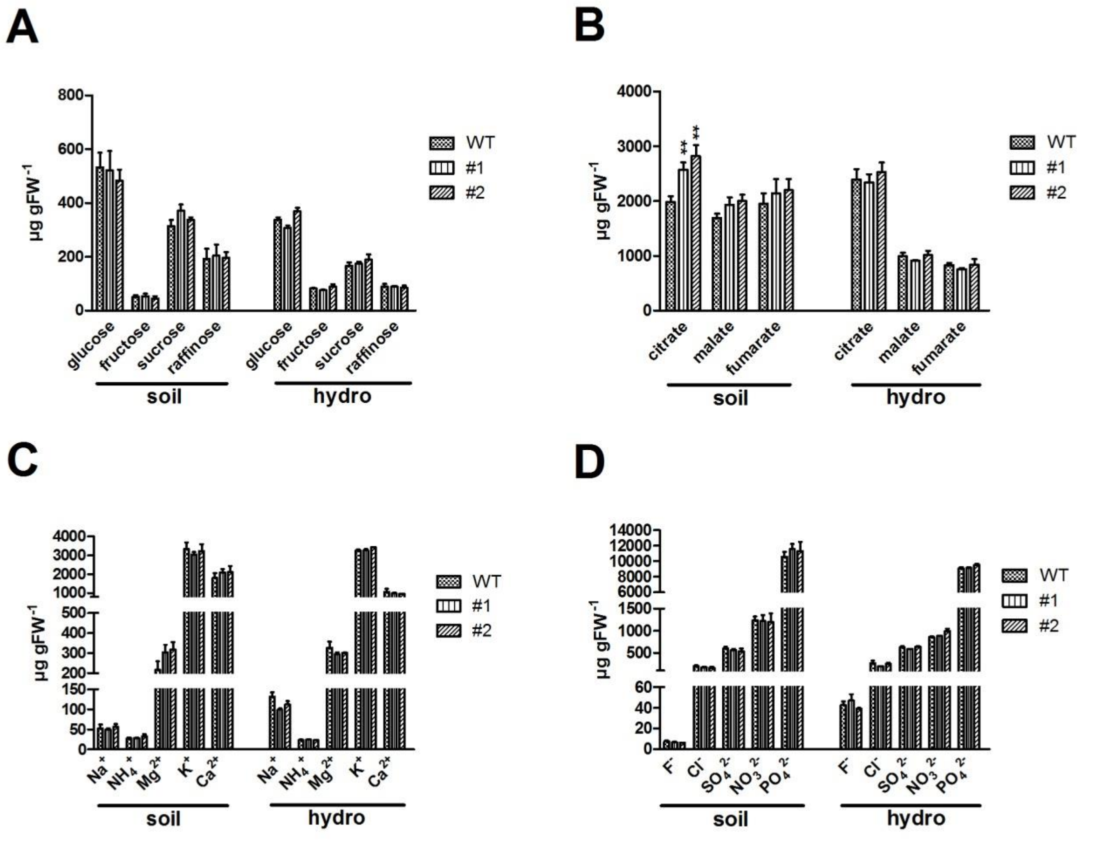

Supplementary Figure 2. Quantification of sugars (A), carbonic acids (B), anions (C) and cations (D). Metabolites were determined in leaves of 4 week old soil or hydroponically grown plants. Data represent means ±SE of four biological replicates. The asterisks indicate differences compared to the control based on Student's t-test (**P < 0.01).
