# Supplementary material for: Apoplastic Nucleoside Accumulation in Arabidopsis Leads to Reduced Photosynthetic Performance and Increased Susceptibility Against Botrytis cinerea
Source: Front Plant Sci. 2015 Dec 23;6:1158. doi: 10.3389/fpls.2015.01158 (PMC4688390; doi:10.3389/fpls.2015.01158)
Supplement: Supplementary file 3 [file Image3.pdf]

Supplementary Material

Apoplastic nucleoside accumulation in Arabidopsis leads to reduced photosynthetic performance and increased susceptibility against *Botrytis cinerea*.

Manuel Daumann, Marietta Fischer, Sandra Niopek-Witz, Christopher Girke, Torsten Möhlmann*

*** Correspondence:** Corresponding Author: Dr. Torsten Möhlmann

email: moehlmann@biologie.uni-kl.de

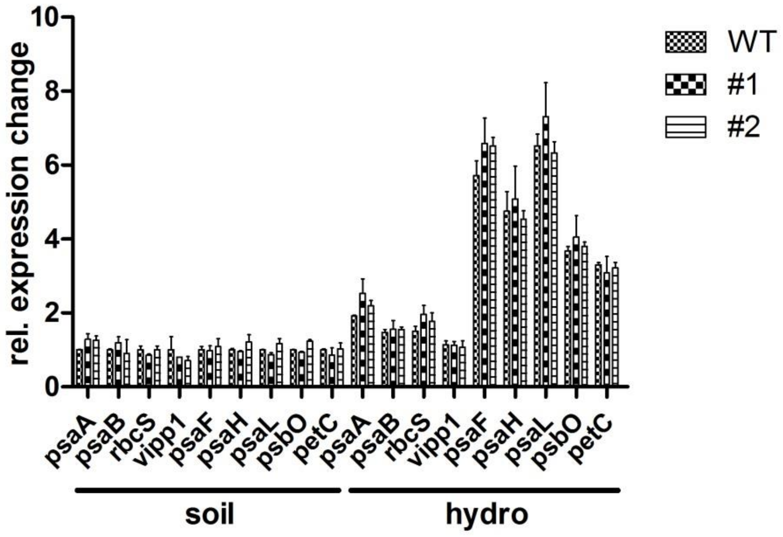

Supplementary Figure 3. Expressional analysis of photosynthesis related genes. RNA was isolated from 3 week old WT and ent3:nsh3 double KO plants grown under ambient conditions on soil or hydroponic culture. The expression of PSAA, PSAB, PSAF, PSAH, PSAL, PSBO, PETC, RBCS and VIPP1 was normalized using the geometric meridian of the housekeeping genes GAPDH, ACTIN and 18SRNA. Data represent means ±SE of three biological replicates.
