# Supplementary material for: Apoplastic Nucleoside Accumulation in Arabidopsis Leads to Reduced Photosynthetic Performance and Increased Susceptibility Against Botrytis cinerea
Source: Front Plant Sci. 2015 Dec 23;6:1158. doi: 10.3389/fpls.2015.01158 (PMC4688390; doi:10.3389/fpls.2015.01158)
Supplement: Supplementary file 4 [file Table1.DOCX]

Supplementary Material

**Apoplastic nucleoside accumulation in Arabidopsis leads to reduced photosynthetic performance and increased susceptibility against *Botrytis cinerea*.**

**Manuel Daumann, Marietta Fischer, Sandra Niopek-Witz, Christopher Girke, Torsten Möhlmann***

*** Correspondence:** Corresponding Author: Dr. Torsten Möhlmann

email: moehlmann@biologie.uni-kl.de

Supplementary Tables

**Supplementary Table 1.** Primers used in this study

| *Gene* | s-sequence [5´🡪3´] | as-sequence [5´🡪3´] |
| --- | --- | --- |
| knockout screening | | |
| *NSH3* | GGAATGTCAACCACGACAGTG | GCACTCTTCTTGATGTCAAATCC |
| *ENT3* | GTTCTTTGGATCCCAATGGCGGATAGATATG | TGAACCACTGTTCTCGAGTGATTAAAACTC |
| *T-DNA LB3* | TAGCATCTGAATTTCATAACCAATCTCGATACAC |  |
| *T-DNA LB335* | ACTCAACCCTATCTCGGGCTATTC |  |
| *RT-ENT3* | GGTCCGGAGCAGAATGCGTT | GGTTACAAGCAAAGAATATGAAAGC |
| *RT-NSH3* | GGTGTTCCAGTGACTCTGG | AGCCGTGAAAGAATCCCAC |
| housekeeping genes | | |
| *ACTIN2* | CTTGCACCAAGCAGCATGAA | CCGATCCAGACACTGTACTTCCTT |
| *EF1α* | GAGACCACCAAGTACTACTGCAC | GTTGGTCCCTTGTACCAGTCAAG |
| *GAPDH* | TTGGTGACAACAGGTCAAGCA | AAACTTGTCGCTCAATGCAATC |
| *UBQ10* | GGCCTTGTATAATCCCTGATGAATAAG | AAAGAGATAACAGGAACGGGAAACATAGT |
| *18SRNA* | ACTTATGGAAGGGACGCATTT | CATCCCAAGGTTCAACTACGA |
| photosynthesis related genes | | |
| *DPT1* | TTACCTCTTGCTCCCCAATCT | TCTCTCCTTTGATTGCTTTGG |
| *PETC* | GATGGCGATGTCAAGTGG | GCTTCATCTATATCCGCGTG |
| *PSAA* | AGGCTTCCACAGTTTTGGTTT | CCCAAACATCTGACTGCATTT |
| *PSAB* | ACCCCGACTCGAGTAGTCATT | CGCAGCTTGAGTCGTAAAATC |
| *PSAC* | GAGCATGCCCTACAGACGTA | TTCGAGTTGTTTCATGCCATA |
| *PSAD* | ATGGCAACTCAAGCCG | CTCTTCCTGGATTCGCTTTC |
| *PSAF* | GCAAGGACTCAAAACAGTTCG | AACTCTCTGTAGGCAGCAACG |
| *PSAH* | ATGGCGTCTCTTGCAACC | TCCTGAGGACCTCTCTTGATG |
| *PSAL* | CGAGCTGTTAAATCCGACAAG | GTGAACTTAGCCCATCCATCA |
| *PSBA* | CTTCTGCAGCTATTGGATTGC | CATTTTCTGTGGTTTCCCTGA |
| *PSBO* | CTGCTTCGAGCCTACTTCCTT | GCAGTGTTCTTCACGTTCTCC |
| *RBCL* | GCGTATGTAGCTTATCCC | TCCCCCTGTTAAGTAGTC |
| *RBCS* | CACTATGGTCGCTCCTTTCAA | GTGAAGCTTGGTGGCTTGTAG |
| *VIPP1* | TCTTGCACGTGAGGCCCTTAAAC | TTCAAAGCAGTAGCGTTGTCAGC |
| pathogenesis related genes | | |
| *ATRBOHF* | AGCAGAACGAGCATCACCTT | GGATTCGATCTCGGATTTCA |
| *BcACTIN* | TCTGTCTTGGGTCTTGAGAG | GTGCAAGAGCAGTGATTTC |
| *JMT* | AGAATCGTGTTTTGTGTCGGCC | GATAAAACCATTCGGCCTCCCG |
| *ORA59* | AGGCAGCCTCGCAGTACTCAA | CTCTTCAAGGCTATCACCGGA |
| *PAD3* | CCGGTGAATCTTGAGAGAGCC | GATCAGCTCGGTCATTCCCC |
| *PDF1.2* | CCATCATCACCCTTATCTTCGC | GTAACAGATACACTTGTGTGCTGG |
| *PR1* | GGCCTTACGGGGAAAACTTAG | GGCTTCTCGTTCACATAATTCCC |
| *WRKY33* | ACGTCATCATGCTCACAGGTTCAG | TTCTTTGCCTGGCTCTTCCTTCTC |
| uptake experiments | | |
| *W303-ENT3s* | TTGAATTCATGGCGGATAGATATGA | TTGAATTCTCAAAAGGCATTCTTCTTAC |
